# Pilot testing a novel remotely delivered intensive outpatient program for hospitalized patients with opioid use disorder

**DOI:** 10.1186/s13722-025-00589-4

**Published:** 2025-08-01

**Authors:** Veronica Szpak, Andrea Velez, Sara Prostko, Naomi Rosenblum, Rie Maurer, Lyndon J. Aguiar, Roger D. Weiss, Joji Suzuki

**Affiliations:** 1 Department of Psychiatry, Brigham and Women’s Hospital, 60 Fenwood Rd, Boston, MA 02115 USA; 2 Harvard Medical School, 60 Fenwood Rd, Boston, MA 02115 USA; 3 Williamsville Wellness, 10515 Cabaniss Ln, Hanover, VA 23069 USA; 4 McLean Hospital, 60 Fenwood Rd, Belmont, MA 02115 USA; 5 Harvard Catalyst, Boston,, MA USA

## Abstract

**Background:**

Individuals with opioid use disorder (OUD) are frequently hospitalized for injection-related medical complications, yet they often receive inadequate treatment for the OUD itself. We previously conducted a qualitative study to adapt an existing remotely delivered intensive outpatient program (IOP) specifically for hospitalized patients with OUD. We then conducted a pilot feasibility and acceptability study to assess the program.

**Methods:**

The 4-week IOP consisted of asynchronous video content and in-person peer support. The primary outcomes were the feasibility of recruitment, acceptability of the treatment as assessed by the completion of videos, and engagement with the peer recovery coach. Secondary outcomes included OUD treatment retention.

**Results:**

Of the 12 participants, the mean age was 40.9 years, 58.3% were female, and 58.3% had an injection-related serious infection. Results demonstrated potentially acceptable recruitment feasibility (70.6%, 95% CI [48.9–92.3]), but the median percentage of video completion was only 2% (range: 0–16%) and the median percentage of engagement with recovery coach was 31.8% (range: 16.7–66.7%). All participants received medications for OUD (MOUD) during the hospital stay (methadone 83%, buprenorphine 17%), and 33.3% remained retained in MOUD treatment at 28 days.

**Conclusions:**

Hospitalized patients with OUD desired additional support through an IOP along with MOUD. While recruitment feasibility was acceptable, the overall program was not. Future research should explore IOP content that is more personalized and engaging while also including peer support.

## Background

A significant risk associated with injection drug use in individuals with opioid use disorder (OUD) is the potential to develop serious injection-related infections, such as infective endocarditis, spinal abscesses, and osteomyelitis [1, 2]. Hospitalizations related to these infections have doubled in the US in the last two decades, with some states experiencing a six-fold increase [3–5]. Individuals with these life-threatening infections are often required to be hospitalized for 6 weeks or more to receive intravenous antibiotics and sometimes surgery, such as cardiac surgery to repair infected heart valves. Despite these efforts, in-hospital mortality approaches 10%, patient-directed discharge occurs in 20% of cases, and 1-year mortality can approach 30% [6–9]. While most hospitals can diagnose and treat these severe infections with appropriate evidence-based medical and surgical interventions, the underlying OUD that precipitated the infection in the first place often goes unaddressed [10]. To address this need, some hospitals have implemented addiction consultation services staffed by addiction specialists to aid hospital teams in the initiation of MOUD, linkage to addiction treatment, and provision of peer support [11–13]. There is a robust evidence-base demonstrating the substantial reduction in mortality rate from overdoses for OUD patients engaged in MOUD treatment [14, 15]. Individuals recovering from these serious infections also benefit greatly from the initiation of MOUD by helping to reduce overdose events [5, 9]. However, studies show that these patients have poor mortality outcomes—in a study of every individual with injection-related infective endocarditis hospitalized in Massachusetts between 2011 and 2015, mortality at 1-year after discharge was no different among those who did and did not initiate MOUD [7]. These data suggest that patients with OUD who are recovering from serious infections are at particularly high risk, not only from treatment discontinuation but also repeat infections that then contribute to the high mortality rate.

Because these patients must be hospitalized for 6 weeks or more, waiting to offer addiction treatment until hospital discharge may represent a missed opportunity. The time could be used to offer more intensive addiction treatment concurrently with the initiation of MOUD given that drug counseling can improve outcomes in OUD patients [16]. Unfortunately, most hospitals do not have an addiction consultation service, and many hospitalized patients have difficulty accessing addiction treatment programs due to having an inconsistent and unpredictable schedule involving tests and procedures that can occur at various times throughout the day. As such, hospitalized patients with OUD who could benefit from a more robust addiction treatment program have very few, if any, options available to them. involving.

Therefore, a remote intensive outpatient program (IOP) that combines synchronous and asynchronous elements could be offered to meet this need. IOPs have robust empirical support in reducing substance use comparable to inpatient or residential treatment [17, 18]. However, IOPs typically offer nine hours or more of structured individual and group treatment per week, where patients learn early-stage relapse management and coping skills, and address problems related to psychosocial well-being [18]. Since hospitalized patients have unique needs different from outpatient treatment seekers, we opted to adapt an existing remotely delivered IOP (“Smart IOP”), which could be completed asynchronously on a mobile device, but attempt to re-create the intensity and content of traditional IOPs. For instance, Smart IOP allows patients to watch recovery-related videos on their mobile device at their own pace and meet a recovery coach weekly to monitor the patient’s progress in the program. The flexibility in how the treatment can be completed suggests that the remote IOP may be particularly appropriate for hospitalized patients, as a traditional IOP may disrupt their medical care due to the synchronous nature of these IOPs.

We conducted a pilot study to test a remotely delivered IOP tailored to the unique needs of the hospitalized OUD patient population. We previously reported on the procedures we underwent to adapt and develop the IOP by conducting a qualitative study to obtain feedback from patients who had survived a hospitalization for serious infections due to injection drug use [19]. Here we report on the pilot feasibility trial we conducted using the newly tailored IOP. We additionally aimed to evaluate the preliminary efficacy of this treatment by examining OUD-related outcomes after discharge from the hospital.

## Methods

### Setting

This research study was conducted at Brigham and Women’s Hospital, a large academic medical center in Boston, on the inpatient medical floors between November 2023 and June 2024. The study received approval from the Mass General Brigham IRB and was registered on ClinicalTrials.gov (NCT05817825).

### Study design

This is a Stage 1b pilot feasibility trial as defined by the NIH Stage Model for Behavioral Intervention Development.

### Participants

Inclusion criteria were adults aged 18 years or older, a diagnosis of DSM-5 severe OUD, and hospitalized for a serious injection-related infection. Exclusion criteria included having active psychosis, suicidality, homicidality, a condition likely to be terminal during the study period and being unable to perform consent due to impaired mental status. However, over the course of the trial, the decision to expand enrollment to those with any DSM-5 substance use disorder without infections was made, given that most patients with serious infections were no longer remaining in the hospital but were being discharged home with oral or long-acting injectable antibiotics.

The addiction consultation team members initially alerted the study staff of potential participants from the hospital inpatients who were seen for consultation. Then the research staff approached potential participants after receiving permission from one of the clinical staff treating the patient. Participants were compensated up to $100: $50 for completing the Smart IOP (SIOP) and $50 for the remote 28-day follow-up, which was through a phone call.

### Smart intensive outpatient program

Smart IOP is a remote IOP that was originally developed in 2016 for individuals in rural Virginia to access addiction treatment remotely. The program was designed to be completed over 8–12 weeks, consisting of asynchronous video modules that patients could watch at their own convenience and time. The program required periodic live sessions with a therapist and case managers. Additionally, patients needed to have a “smart sponsor,” usually a family member or friend, to provide support and confirm abstinence to the case managers and therapist.

### Adaptation of SIOP

**Fig. 1:**
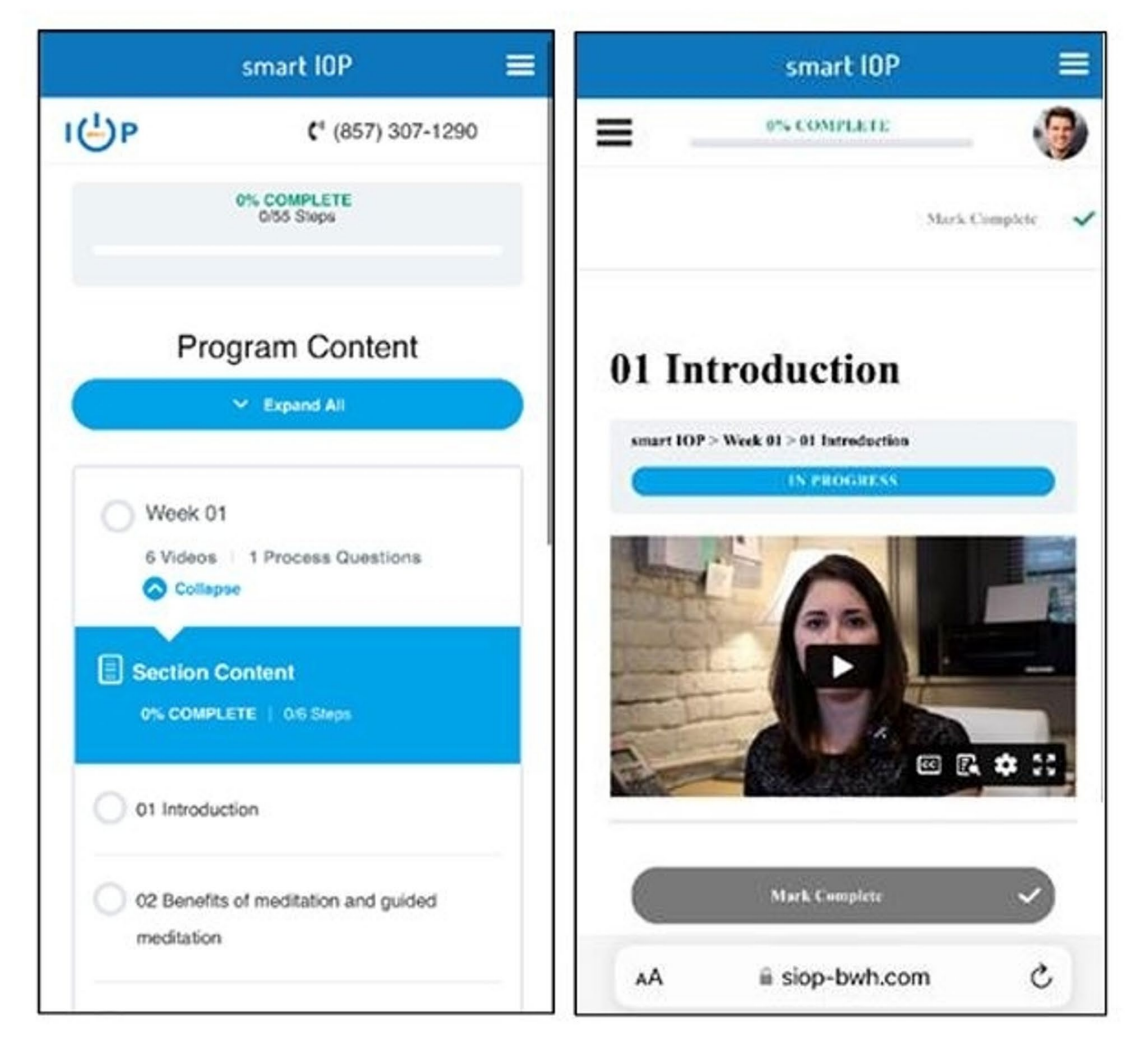
This figure shows an example of the participants’ view of the Smart IOP App

The original program was adapted to better meet the needs of hospitalized patients. A qualitative study was conducted with individuals with OUD who had survived an injection-related infection to obtain their feedback on content and potential benefits of the SIOP [19]. Briefly, respondents agreed that an online addiction treatment program may indeed be beneficial, and that both peer support and aftercare planning would be important elements to include. Adaptations were made based on their feedback, including adding additional video content to address the challenges unique to hospitalized patients. The duration of the program was also shortened, allowing for the program to be completed in 4 weeks during their inpatient stay. However, if a participant was discharged early, then they were expected to continue the program at home. As part of these modifications, a peer recovery coach fulfilled the role of the “smart sponsor,” instead of a family member or friend. The peer recovery coach offered both in-person and remote support for the duration of the study. See Fig. 1 of the Smart IOP App.

### Study procedures

After giving informed consent, participants completed the following baseline measures: Timeline follow-back (TLFB) [20] to assess the past 28-day use of substances using a calendar method; Brief addiction monitor (BAM) [21] to assess substance use, risk factors, and protective factors related to physical health, alcohol and drug use; and the Opioid craving scale (OCS) [22] to assess opioid craving. A high score on BAM indicates more protective or risk factors, and a low score indicates fewer protective or risk factors. A high score in the OCS indicates a high level of craving, and a low score indicates a low level of craving. The research team then assisted the participants in setting up and using the SIOP on their device; a tablet device to access the program was provided free of charge if the participant preferred. Participants were invited to watch the videos of their choosing, starting with an introductory video. The program included a curated content of 40 videos, each 5–45 min long, addressing topics such as the nature of addiction, coping strategies for cravings, relapse prevention, mindfulness, depression management, relationships, and coping with prolonged hospitalizations. Videos could be watched in any order at any time of the participant’s choosing. Participants were also introduced to the peer recovery coach and were reminded that they would meet with the peer recovery coach weekly, in-person or remotely. During these weekly visits, the peer recovery coach: (1) encouraged completion of the program, (2) answered any questions participants had about the program, and (3) reinforced the importance of aftercare to continue addiction treatment after discharge from the hospital. For each participant, the study team recorded the number of video modules completed each week, as well as whether there was engagement with the recovery coach via texting, phone, or in-person meetings.

### Follow-up assessments

Initially, follow-up assessments were scheduled after participants were discharged from the hospital. However, due to participants’ being discharged sooner than anticipated and to a protocol change that permitted the enrollment of those without serious infections, follow-up assessments were scheduled starting after the completion of the 4-week program.

### Primary outcomes

Our primary outcomes were to evaluate the feasibility of recruitment and acceptability of the program in completing the video content. A priori, we established a benchmark for recruitment feasibility as “acceptable” if at least 70% of those eligible agreed to participate [23]. We also established a benchmark for program acceptability as “excellent” if participants watched at least 70% of the video content, or “good” if they completed at least 60% of the videos. Post-hoc, we also chose to assess engagement with the peer recovery coach using similar benchmarks: “excellent” if participants engaged with the coach in at least 70% of the possible days, and “good” if they engaged with the coach in at least 60% of the possible days.

### Secondary outcomes

After completion of the program, participants remotely completed a 7-, 14-, 21-, and 28-day follow-up assessments with the following measures: TLFB, OCS, BAM (see above for description), and engagement with MOUD and other treatments such as residential programs, IOPs, or attending mutual-help meetings.

### Statistical analysis

Descriptive statistics were used to summarize the results. The primary outcome of recruitment feasibility and program acceptability were reported with proportion and 95% confidence intervals. We prespecified the upper boundary of the unacceptable zone to be 40%. A Z-test for one proportion was conducted and a one-sided *p*-value was reported.

## Results

### Participants

**Fig. 2:**
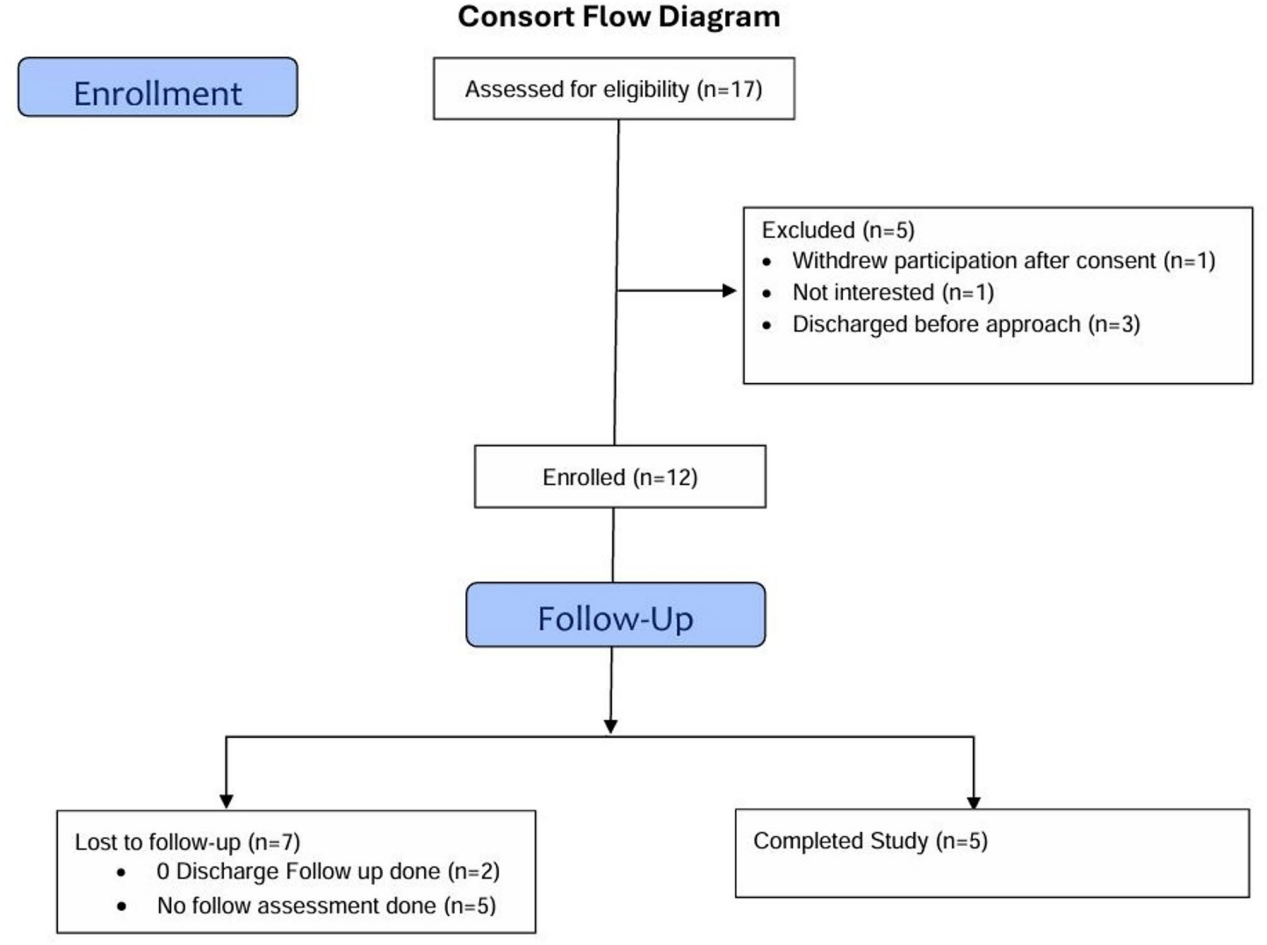
This consort diagram illustrates the enrollment of particpants and their progress in the study, including lost to follow-up and completion

A total of 17 particpants were assessed for eligibility, and five were excluded (see Fig. 2 for the consort diagram). Twelve participants (70.6%) successfully enrolled in the trial. The mean age was 40.9 years, 58.3% were female, and 58.3% had an injection-related serious infection. Additional participant characteristics are presented in Table 1.

**Table 1:** Baseline characteristics of participants

Participant Characteristics	*N* = 12
Age (SD)	40.9 (8.65)
Sex, Female, n (%)	7 (58.3%)
Race, White, n (%)	11(91.7%)
Ethnicity, non-Hispanic, n (%)	11(91.7%)
Substances, n (%)	
Opioid Use Disorder	12 (100%)
Alcohol Use Disorder	3 (25%)
Cocaine Use Disorder	6 (50%)
Stimulant Use Disorder	6 (50%)
Cannabis Use Disorder	1 (8.3%)
Benzodiazepine Use Disorder	3 (25%)
Tobacco Use Disorder	6 (50%)
Infection Status, n (%)	
Injection Related Infection	7 (58.3%)
Endocarditis	2 (16.7%)
Osteomyelitis	2 (16.7%)
Abscess	1 (8.3%)
Septic Arthritis	1 (8.3%)
Bacteremia	2 (16.7%)
Medication for Opioid Use Disorder during Hospitalization, n (%)	
Methadone	10 (83.3%)
Buprenorphine	2 (16.7%)

### Recruitment feasibility

The recruitment feasibility of this program was significantly higher than the prespecified unacceptable feasibility upper boundary of 40% (*p* = 0.005). Therefore, the feasibility of 70.6% (95% CI 48.9–92.3) falls in the potentially acceptable range.

### Video and peer recovery coach engagement

The median percentage of video completion among 12 participants was 2% (Range: 0–16%). The engagement with the peer recovery coach is summarized in Table 2. Participants could meet with the peer recovery coach three times per week and were expected to engage at least once a week. The median engagement with the recovery coach was 31.8% (Range: 16.7-66.7%).

**Table 2:** Frequency of recovery coach engagement with participants

Recovery Coach Engagement, Median (Range)	
Total Number of Weeks	4.0 (1-11)
Total Number of Sessions in Days	2.5 (1-10)
Engagement Percentage	31.8% (16.7-66.7)
Total Number of Engagement in Minutes	55 (15-260)
Total Number of Phone Calls/Texts in Minutes	20 (0-64)
Total Number of In-Person Minutes	30 (0-205)
In-Person Engagement Percentage	72.8% (0-100)

## Secondary exploratory outcomes

### Addiction-related outcomes

All participants received MOUD during the hospital stay: 83.3% received methadone and 16.7% received buprenorphine. Four out of five participants continued to engage in MOUD treatment after discharge. If we assume that all seven participants lost to follow-up experienced a “relapse,” then 33.3% of participants remained retained in MOUD treatment at the 28-day follow-up.

### Brief addiction monitor

The total protective factors mean (± SD) score at baseline (*n* = 12) was 49.3 ± 19.6, and 81.8 ± 50.0 at the 28-day follow-up (*n* = 5). The total risk factors mean (± SD) score at baseline was 101.5 ± 36.1 and 64.2 ± 31.7 at the 28-day follow up. The total (substance) use score at baseline was 25.3 ± 26.8 and 22.0 ± 33.3 at the 28-day follow up.

### Timeline follow-back

The mean ± SD proportion of days (over 28 days prior to baseline assessment) that participants used opioids, heroin, or fentanyl before beginning the IOP was 63.6% ±35.6. Three patients reported using opioid analgesics, including methadone/heroin/fentanyl, during the study period since discharge.

### Opioid craving scale

The mean ± SD craving score at baseline (*n* = 12) was 4.8 ± 3.0 and 3.2 ± 3.1 at the 28-day follow-up (*n* = 5).

## Discussion

This study aimed to examine the feasibility and acceptability of a 4-week, remotely delivered, asynchronous IOP for hospitalized patients with OUD, focusing on engagement with video modules and a peer recovery coach. The recruitment feasibility was potentially acceptable; however, the video engagement was low and engagement with the peer recovery coach was moderate. Consequently, the program was not considered acceptable. These results point to hospitalized patients’ willingness to consider completing an IOP remotely with additional support from a peer recovery coach, and that focusing on peer support may be more effective than watching videos passively.

Overall, the videos did not seem to be sufficiently engaging or personalized. This could be an area of improvement in future studies involving treatments that utilize asynchronous content. This also indicates that more research is needed to understand how to adapt technology-based interventions for hospitalized patients with OUD. One way to increase participant engagement in future studies could include contingency management, which has been suggested by recent reseach [24].

While participants could engage with the recovery coach up to three times per week, in-person and remote contact was limited. The participants expressed interest in meeting with the recovery coach; however, there were instances such as undergoing testing during the recovery coach’s visits that made it difficult to meet. In addition, their weakened physical and mental state due to the serious infection made it challenging for them to engage. This underscores the lack of resources available to this patient population. There is indeed emerging evidence to suggest that consistent engagement with a peer recovery coach may improve MOUD retention rate. Another study of hospitalized patients with alcohol use disorder found that those who engaged with a peer recovery coach had higher rates of treatment post-discharge linkage [25]. Furthermore, another study found that engagement with a peer recovery coach increased the odds of buprenorphine treatment in patients with OUD [26].

There are limitations to this study. This was a pilot study with a small sample size and used a convenience sample. In addition, our sample was homogeneous in terms of demographics (mainly white and non-Hispanic), which makes it difficult to generalize to the general population. Half of our particpants were also lost to follow-up by the 28-day follow-up period. Although we made every effort to follow up with our participants post-discharge, we faced challenges such as particpants lacking access to a cell phone.

## Conclusions

While video engagement was low, our results indicate that further research is needed to determine if technology-based interventions are suitable for this hospitalized patient population with a comorbid serious medical illness. Additionally, more research is necessary to evaluate the effectiveness of peer recovery coach interventions in improving OUD-related outcomes.

## Data Availability

The datasets used and/or analyzed during the current study are available from the corresponding author on reasonable request.

## Declarations

### Clinical trials registration

NCT05817825.
