# Correction: Pilot testing a novel remotely delivered intensive outpatient program for hospitalized patients with opioid use disorder

**DOI:** 10.1186/s13722-025-00601-x

**Published:** 2025-09-08

**Authors:** Veronica Szpak, Andrea Velez, Sara Prostko, Naomi Rosenblum, Rie Maurer, Lyndon J. Aguiar, Roger D. Weiss, Joji Suzuki

**Affiliations:** 1 Department of Psychiatry, Brigham and Women’s Hospital, 60 Fenwood Rd, Boston, MA 02115 USA; 2 Harvard Medical School, 60 Fenwood Rd, Boston, MA 02115 USA; 3 Williamsville Wellness, 10515 Cabaniss Ln, Hanover, VA 23069 USA; 4 McLean Hospital, 60 Fenwood Rd, Belmont, MA 02115 USA; 5 Harvard Catalyst, Boston,, MA USA

**Correction:** ***Addict Sci Clin Pract*** **20, 61 (2025)**

10.1186/s13722-025-00589-4

Following the publication of the original article [1], it is reported that a co-author’s name was incorrect due to the acronym for her certification being incorrectly captured as her family name. “Andrea Velez Carc” has been corrected to “Andrea Velez”.

The original article [1] has been updated.
